# Notes and Notices

**Published:** 1892-09

**Authors:** 


### NOTES AND NOTICES.

Dr. J. H. McClelland, the new President of the American Institute of Homœopathy, was born in Pittsburgh, Pa., of Scotch-Irish parentage, some forty seven years ago. His preliminary education was secured in the Western University, after which he became a disciple of Homœopathy in the Hahnemann Medical College, of Philadelphia, graduating in 1867. He immediately established himself in Pittsburgh, where he has been a successful practitioner ever since. He was actively instrumental in establishing the Homœopathic Hospital in that city some twenty-five years ago, and as a member of its surgical staff has advanced the interests and fame of the institution materially. Dr. McClelland is a quiet, unostentatious gentleman, but possessed of great executive ability. That his merits have been appreciated, however, is proved by the fact that he is President of the State Board of Health, of Pennsylvania, a member of the American Public Health Association, and the American Association of Social and Political Science. Last, but not least, he has just been elected, without the slightest effort or campaigning on his part, President of the American Institute, of which he is also a Senior of over twenty-five years' membership.—*Washington Post.*

Homœopathy in the United States.—Hans Birch Gram was born in Boston in the last century. He was the son of Hans Gram, of Copenhagen, where after the death of his father he was educated. He was the first to introduce Homœopathy to the United States. He finished his medical studies in 1814 with honor, and in the year 1823, after having investigated Homœopathy or the doctrine of Hahenemann, was converted and became a fervent defender of the same.

He went to New York in 1825, where he obtained splendid and numerous results, converting a great number of doctors, and although his school was combatted by the allopaths with all kinds of weapons, the new system of medicine spread in other cities in New York State, and in many other States of the Union.

Dr. Hering, a disciple of Hahnemann, introduced the reform into the State of Pennsylvania, where in a short time he made numerous proselytes.

The increase of the homœopathic system was extensive. An increase that was certain and sure, notwithstanding the opposition and insults of the old school, so that at present the laws of almost all the States concede to the homœopathist the same rights and privileges that the allopaths have.

"Actually," says Dr. Wells, "we have a Homœopathic College in New York, one in Philadelphia, one in Baltimore, one in Cincinnati, two in Cleveland, two in Chicago, one in St. Louis, one in Ann Arbor, Michigan, one in Detroit, one in Iowa City, and one in San Francisco. In all of these cities they have good dispensaries and hospitals."

New York has three hundred and fifty homœopathic physicians, in Chicago, three hundred and fifty-six. In Philadelphia and other cities numbers in porportion to their populations. The homœopathic physicians in the United States number about ten thousand.

The American Institute of Homœopathy, a voluntary association of the physicians of each State, was founded in 1844, and now has a thousand members. One thing worthy of attention is that those who were first converted in the old school have remained faithful to Hahnemann as much in practice as in principle, meanwhile many of those of the new-comers have turned eclectics, and these can also, as any other person who possesses a medical diploma, become a member of the Institute. It followed in consequence that in each reunion till in 1874 these eclectics were very numerous, so a separation became necessary, which resulted, in 1880, in the founding of The International Hahnemannian Association, which only accepts physicians who follow faithfully the doctrines of Hahnemann. The actual members are nearly two hundred.

As is always the case, Spain is relegated to oblivion, although in relation to its population it is very probable that it has more representatives of Hahnemann's principles than some of the other countries mentioned by Dr. Wells. We do not wish to accuse any one, but we must acknowledge that we have been indolent in not having made plain our affiliation with the large family of Hahnemann. It is now more necessary than ever that we enumerate ourselves, showing to the world our value in contradiction of this opinion, and how firm and faithful we are to the pure principles practiced by the immortal sage.—*Revista Homœopatica de Barcelona,* Enero, 1892.

Dr. Rufus Leander Thurston has removed to Hotel Hamilton, corner Clarendon Street and Commonwealth Avenue, Boston.

A. L. Kennedy, M. D., has removed to Hotel Hamilton, corner Clarendon Street and Commonwealth Avenue, Boston.

Dr. F. L. Griffith, of Edina, Missouri, will move to Austin, Texas, about August 30th, where he has formed a partnership with Dr. Thomas H. Bragg. This will be a purely homœopathic combination.

Dr. Pemberton Dudley, General Secretary of the American Institute of Homœopathy, will remove about August 1st, to No. 1405 North Sixteenth Street, Philadelphia.

Dr. Ida Wright Rogers, Editor of the *People's Health Journal,* and Professor of Dietetics and Personal Hygiene in the National Homœopathic College, of Chicago, arrived in Liverpool August 18th, and will spend several months abroad in study and sight-seeing.

Think of it! The New York Homœopathic Medical College and Hospital announces in its circular for the ensuing year: "Model prescriptions will be made, at least, once a week." Query. What kind of prescriptions are the remaining ones? Hitchcock.

The Hering College of Homœopathy is the name of a new College to be started in Chicago in October. In their circular, the projectors say: "In announcing the organization and establishment of the Hering College of Homœopathy, those responsible for its existence hardly feel that an explanation is demanded beyond the statement that the institution is to teach the philosophy of Homœopathy and the facts of the Homœopathic Materia Medica. It is true that there are a great many medical colleges in this country, some of which are homœopathic in name, but there is not one college in America or in the world that thoroughly, courageously, and persistently teaches certain truths which we hold to be as essential as they are eternal. Upon the truths of the *Organon* of Samuel Hahnemann, as illustrated and confirmed in practice by men such as Dunham, Farrington, Lippe, Hering, and Guernsey, is founded the Hering College of Homœopathy, and we submit the justice of our plea and the uprightness of our purpose to those who believe that the appropriate place to test medical theories is the sick room. * * * As we believe the first and highest duty of the physician to be the restoring of health to the sick, we shall devote extraordinary time and effort to the teaching of the facts of the Homœopathic Materia Medica and the principles governing their practical application, for nearly all young practitioners fail because they have not been taught how to apply the remedy." For further information address L. A. L. Day, M. D., 70 State Street, Chicago.

WANTED.—Volumes II and V of Hahnemann's *Chronic Diseases*, Hempel's translation. Or a complete set of the *Chronic Diseases* will be bought if a reasonable price be asked. Wanted also volumes I, II, and III of THE HOMŒOPATHIC PHYSICIAN. For sale or exchange one copy Hering's *Analytical Therapeutics*, Vol. I, cloth, one copy Gross' *Comparative Materia Medica*, half morocco, one copy Grauvogel's *Text Book of Homœopathy*, half morocco.

All these books are good; all are rare, all in good order.

Apply to S. C. care of THE HOMŒOPATHIC PHYSICIAN, 1125 Spruce St., Philadelphia, Penna.

DR. GEORGE WILLIAM WINTERBURN, editor of the *Homœopathic Journal of Obstetrics, Gynecology, and Pedology*, has removed to his new house, No. 230 West 132d Street, New York.

### WORLD'S CONGRESS NOTES.

The decision of the American Institute to hold its next session in connection with the World's Congress of Homœopathy at Chicago, in 1893, will insure the largest and most representative meeting of our school ever held.

The International Hahnemannian Association has been invited to take part in the Congress.

The Great Northern Hotel—new and elegantly furnished—absolutely fireproof, has been engaged for the headquarters of the Congress. It is about three blocks from the Art Building where the sessions of the Congress will be held. Rooms will be furnished at regular rates. Application should be made at once to Dr. J. H. Buffum, Venetian Building, Chicago.

The magnificent Art Building, to cost $1 000,000, in which the meetings of the Congress are to be held, is now being rapidly built, and will be completed May 1st, 1893. It will contain two audience rooms seating 3,500 each and a dozen or more halls seating from 300 to 700 each. Ample facilities will be offered for introductory exercises, general sessions, and committee meetings under the same roof.

The new four-mile intake will be ready for use in a few weeks, and Chicago will then have one of the best systems for securing pure water in the world.

One of the most interesting studies for physicians at the Exposition will be the sewerage system. Six thousand sanitary closets will be built in marble compartments. From these the sewerage will be conveyed to large tanks at the southeast corner of the grounds; there purified by chemicals—its solids pressed into cakes and burned in furnaces. Arrangements are made for a permanent city of 300,000 inhabitants. This method will therefore receive a thorough test.

The Congress will convene Monday, May 29th, 1893, and continue its sessions through the week, the last session being held June 3d.

It is hoped that the attractions of the Exposition, with those of the Congress, will secure a large representation of physicians of our school from foreign countries. The Committee will make earnest endeavors to secure such delegates.

### FUN FOR DOCTORS.

Doctor—"My dear madam, there is nothing the matter with you—you only need rest."

"But, doctor, just look at my tongue!"

"Needs rest too, madam."—*Fliegende Blatter.*

An eminent physician recently gave a medical student the following advice: "Never, never send in a bill for odd dollars or cents For instance, suppose my bill for some particular case amounts to $450. Instead of making it out for that amount, I make it an even $500 and—get it. The patient would just as soon pay that, and in nine cases out of ten he will grumble at the former bill and pay the latter without a murmur."

Dangerous Proximity.—"Is your father in immediate danger?"

"Indeed he is. There is a doctor up-stairs now."—*The Jester.*

Polite Waitress—"Tea, doctor?"

Doctor—"No, coffee, if you please."

Waitress—"Roast beef, doctor?"

Doctor—"If you please."

Waitress—"Corn, doctor?"

Doctor (indignantly)—"No, madam, I am a dentist."—*Drake's Magazine.*

Knew His Business.—Mr. Laman—"Why do you always question patients so closely about what they eat? Does the information you get help you to diagnose their cases?"

Doctor Emde—"Oh! no! But by so doing I am enabled to guess what their station in life is, and how much fees I can probably get out of them."

Only Body Snatchers Feared.—Doctor Killem—"Did the medicine I sent your husband cause him to rest easy?"

Mrs. Widderweeds (sadly)—"Yes, unless the medical students have disturbed him."—*Epoch.*

Definition of Apothecary—"A man who mixes drugs of which he knows little, to pour into a body of which he knows less, to cure a disease of which he knows nothing."—*Voltaire.*

